# Editorial: Molecular dissection of the genetic basis underlying plant complex traits

**DOI:** 10.3389/fgene.2023.1256218

**Published:** 2023-07-28

**Authors:** Yuchen Yang

**Affiliations:** State Key Laboratory of Biocontrol, School of Ecology, Sun Yat-sen University, Shenzhen, China

**Keywords:** phenotype, molecular mechanism, epigenetics, DNA methylation, genomics, plasticity

Plant phenotypic divergence is the output of complex regulatory networks (Lachowiec et al., 2016). Taking advantage of recent high-throughput sequencing technologies, pan-genomic and transcriptomic studies allow us to comprehensively investigate the key functional genes and pathways underlying various plant phenotypes of interests (Jayakodi et al., 2021; Lei et al., 2021; Derbyshire et al., 2022). The novel findings will substantially boost molecular-assisted screening and delivery of new crop and fruit cultivars with favorable agronomic traits, which would improve the diversity and safety of our food.

In this Research Topic, Meena et al. summarizes recent discoveries and future perspectives in genomic and genetic mechanisms underlying desirable agronomic traits in sugarcane crops. The alleles and variants revealed can be used as candidate targets for future bioengineering-assisted improvement of sugarcane cultivars to enhance yield.

In another approach, Zhou et al. reviews the classification, evolutionary history and biological function of TCP transcription factors (TFs): TEOSINTE BRANCHED1 (TBI1), CYCLOIDEA (CYC), and PROLIFERATING CELL NUCLEAR ANTIGEN FACTOR1 and 2 (PCF1 and PCF2). TCP TFs are classified into three clades, where the PCF1/2 and CIN clades exist in both lower and higher land plants, while the CYC/TB1 clade emerges relatively later, being only present in eudicot and monocot plants. Sequence divergence further results in functional diversity of TCP proteins, such as regulating plant organ growth and development, phytohormone biosynthesis and abiotic and biotic stress responses. This study presents us an example that highlights the functional importance of TFs in regulating plant morphogenesis. Future work on TFs will provide more resources for plant genetic improvements, especially regarding desired agronomic and adaptive traits.

At the transcriptional level, Meng et al. demonstrates that plant hormones, together with up- and downstream regulatory genes, form a complex regulatory network that modulates the morphological divergence of the leafy head in four types of Chinese cabbage (*Brassica rapa ssp. pekinensis*) cultivars. They show that the growth hormone (auxin) and abscisic acid (ABA)-related genes play key roles in determining the leaf bulb types of Chinese cabbage.

In another example, Li et al. recognizes the gene encoding lipid transfer protein OsLTPL23 as a regulator of seed germination of rice (*Oryza sativa* ssp. *japonica* cv. Nipponbare). The disruption of OsLTPL23 causes abnormal accumulation of endogenous ABA and thus delays seed germination.

In addition to genomic and transcriptomic changes, epigenetic alterations also play important roles in modulating plant phenotypic plasticity (Dar et al., 2022). Wei et al. presents one example where genome-wide changes of the DNA methylation landscape regulate the tolerance to freezing stress in winter rapeseed (*Brassica napus* L.). When exposed to freezing condition, genes encoding the TFs of MYB and bHLH families are found to be differentially methylated, regulating the expression of downstream cold-responsive genes and enhancing freezing tolerance. Furthermore, there are a large number of differentially methylated genes specific to the 17-year-old plants, compared to those of 15 and 16 years. These specific methylation modifications are overrepresented in the signal transduction process of plant hormones, such as jasmonic acid, auxin and ABA, which contribute to explaining the higher overwintering rate of 17-year-old plants. These findings build a link between epigenetic mechanism and phenotype plasticity in plants and also provide valuable resources for future research on genetic improvement of plants with high tolerance to freezing stress.

In conclusion, this Research Topic presents a collection of recent studies on genetic mechanisms modulating plant phenotypic plasticity, at genomic, transcriptomic and epigenomic levels. We hope that readers will find this Research Topic to provide a set of useful references to the field of plant genetics and future bioengineering application in plants.
